# Coexisting Firing Patterns in an Improved Memristive Hindmarsh–Rose Neuron Model with Multi-Frequency Alternating Current Injection

**DOI:** 10.3390/mi14122233

**Published:** 2023-12-12

**Authors:** Mengjiao Wang, Jie Ding, Bingqing Deng, Shaobo He, Herbert Ho-Ching Iu

**Affiliations:** 1 School of Automation and Electronic Information, Xiangtan University, Xiangtan 411105, China; 202221623094@smail.xtu.edu.cn (J.D.); dengbq1997@163.com (B.D.); heshaobo@xtu.edu.cn (S.H.); 2 School of Electrical, Electronic and Computer Engineering, University of Western Australia, Crawley, WA 6009, Australia; herbert.iu@uwa.edu.au

**Keywords:** flux-controlled memristor, magnetic induction, Hindmarsh–Rose neuron model, coexisting firing pattern

## Abstract

With the development of memristor theory, the application of memristor in the field of the nervous system has achieved remarkable results and has bright development prospects. Flux-controlled memristor can be used to describe the magnetic induction effect of the neuron. Based on the Hindmarsh–Rose (HR) neuron model, a new HR neuron model is proposed by introducing a flux-controlled memristor and a multi-frequency excitation with high–low frequency current superimposed. Various firing patterns under single and multiple stimuli are investigated. The model can exhibit different coexisting firing patterns. In addition, when the memristor coupling strength changes, the multiple stability of the model is eliminated, which is a rare phenomenon. Moreover, an analog circuit is built to verify the numerical simulation results.

## 1. Introduction

Neurons are the basic unit of the nervous system structure, and information is transmitted in the nervous system in the form of electrical pulses. The earliest neuron model is the Hodgkin–Huxley (HH) neuron proposed in 1952 [1]. Subsequently, the FitzHugh–Nagumo (FHN) neuron model [2], Morris–Lecar (ML) neuron model [3], HR neuron [4], and Hopfield neural network [5], which are derived from the HH neuron model, were gradually proposed. In recent decades, the dynamic behavior based on various neuron and neural network models has been studied. Among them, HR neurons can effectively simulate the electrical activities in the brain [6]. Many biological neural electrical activity phenomena such as silence, spiking firing, bursting firing, periodic oscillation, and even simple chaos have been verified in the neural model. To study the influence of parameter variation on the firing patterns of neural nonlinear dynamical system, an improved Hindmarsh–Rose neural nonlinear dynamical system model was proposed in [7]. Various firing patterns of neurons can also be observed by changing the initial conditions as discussed in [8], which indicates the memory effect of the neuronal system. The study of firing patterns and chaotic dynamics in neurons has gradually become a popular issue in the international academic community [9].

Furthermore, with the continuous development of memristor theory, the application of memristor in many fields has achieved remarkable results and has bright development prospects [10,11,12,13,14]. It has developed rapidly in the fields of memristor neurons and neural network dynamics. When a neuron is stimulated by electromagnetic radiation, it will accumulate magnetic flux on the cell membrane to generate a magnetically induced current, and the memristor correlates the relationship between magnetic flux and electric charge. Therefore, the flux-controlled memristor can be used to describe the magnetic induction effect, in order to establish a neuron or neural network model affected by electromagnetic radiation. At present, the research on using flux-controlled memristor to describe the electromagnetic induction phenomenon on the cell membrane has gradually emerged. A new three-dimensional memristive HR neuron model is reported in [15]. The HR neuron model can show a discrete memristor initial offset boosting behavior, with an infinite number of discontinuous attractors. By changing the initial value of memristor, the generated firing patterns can be boosted to different discrete levels. In [16], the memristive HR neuron model operates in hidden bursting firing patterns when using an electromagnetic induction current generated by the threshold memristor to replace the external current. The application of locally active memristor in a neuron model has gradually emerged [17,18,19,20]. Ref. [21] proposed a new type of locally active and non-volatile memristor, and it was used as a connecting autapse, so that both the firing pattern and multistability can be observed. Ref. [22] proposed a new neuron model based on a simple structure and excellent characteristics with a locally active memristor, which can exhibit more complex firing behaviors within the locally active region. Ref. [23] coupled 2D HR neurons to a 3D Hopfield neural network using a memristor as a synapse, in which multiple firing patterns and multistability were observed. Additionally, multistable coexistence is common in chaotic systems, and it needs to be eliminated in certain situations [24,25]. For this reason, by introducing a memristor into the HR model, an interesting phenomenon of multistability disappearance can be found when the memristor coupling strength changes, which is rarely reported in previous studies.

For various changes in the internal and external environments of the body, neurons encode, transmit, and decode the information in different firing patterns (the presence or absence of action potential, frequency, peak, etc.). Different external stimuli cause different neural firing patterns and lead to different physiological effects [26,27,28,29]. In fact, a neuronal cell can be thought of as a signal processor that can receive multiple signal inputs at the same time. Strong stimulation can input enough energy to induce mode transition, and the angular frequency can slightly modulate the firing rhythm at a certain intensity. Multiple signal inputs can be equivalent to coherence resonance [30,31]. In addition, the high–low frequency signal is widely used in neuron models. In [32], the mode transition of electrical activities in neurons can be investigated when an electrical stimulation with a high–low frequency current is injected into a new HR neuron model. As discussed in [33], an improved HR neuron model with the periodic high and low frequency electromagnetic radiation and the Gaussian white noise is constructed to explore the modes transition in the electrical activities of neurons. Based on the above ideas, this paper proposed a memristive HR model with multi-frequency AC injection. Under the effect of both the single and double excitation signals, we find that the improved model possesses a property of the coexistence of different firing patterns. In addition, the multistability of the model will be eliminated when the memristor coupling strength *k* changes.

The rest of this paper is organized as follows. An improved memristive HR neuron model with multi-frequency AC injection is constructed in Section 2, and the stability of the equilibrium points is studied. Section 3 discusses the dynamical behaviors under single excitation, and the influence of the memristor coupling strength is also illustrated. Different firing patterns under high–low frequency current are determined in Section 4. The analog circuit is designed in Section 5, and a summary of the full paper is given in Section 6.

## 2. Model Description

In recent years, memristors have developed rapidly in the fields of neurons and neural network dynamics. Memristor neuron network dynamics is a new interdisciplinary subject. When neuron membrane potential is affected by the external magnetic field, induced current will react on the neuron membrane potential. This kind of influence can be described by a flux-controlled memristor [34].

The HR model is a neuron model [4] described by a set of ordinary differential equations with cubic nonlinear terms, and it can be described as

(1) $$\left\{\begin{array}{c} \dot{x}=y+ax^{2}-bx^{3}+I\\ \dot{y}=c-dx^{2}-y \end{array}\right.\;$$

where *x* and *y* denote the membrane potential, and the recovery variable of the neuron, respectively. *a*, *b*, *c*, and *d* are positive parameters. *I* represents the external input current. A simple generalized flux-controlled memristor model is described as

(2) $$\left\{\begin{array}{c} i=W(\varphi )v\\ W\left(\varphi \right)=\alpha +\beta \varphi^{2}\\ \frac{d\varphi }{dt}=v-\varphi \end{array}\right.$$

where *v* and *i* are the input voltage and output currents of the memristor, respectively, φ is the magnetic flux indicated as the state variable, W(φ) is the flux-controlled conductance, and α and β are constant. In this paper, α and β are set to 0 and 0.01, respectively.

By introducing the flux-controlled memristor and injecting two external alternating currents (ACs), an improved memristive HR neuron model with multi-frequency AC injection was constructed as follows

(3) $$\left\{\begin{array}{c} \dot{x}=y+3x^{2}-x^{3}+kW\left(\varphi \right)x+I_{1}+I_{2}\\ \dot{y}=1-5x^{2}-y\\ \dot{\varphi }=x-\varphi \end{array}\right.\;$$

where $I_{1}=A_{1}\sin\left(2\pi f_{1}t\right)$, $I_{2}=A_{2}\sin\left(2\pi f_{2}t\right)$, parameters a=3, b=1, c=1, d=5, and *k* is the memristor coupling strength. *x* and *y* denote the membrane potential and the recovery variable of the neuron, respectively.

The equilibrium point of the proposed improved memristive HR neuron model can be obtained by setting the left side of the Equation (3) as equal to zero

(4) $$\left\{\begin{array}{c} 0=y+3x^{2}-x^{3}+kW\left(\varphi \right)x+I_{1}+I_{2}\\ 0=1-5x^{2}-y\\ 0=x-\varphi \end{array}\right.$$

From $1-5x^{2}-y=0$ and x−φ=0, we know that $y=1-5x^{2}$, x=φ. Next, let x=η and the equilibrium point be set to *E*. Thus, the equilibrium point can be given by

(5) $$E=(\eta ,1-5\eta^{2},\eta )$$

Then, we can substitute *E*, α=0, and β=0.01 into $y+3x^{2}-x^{3}+kW\left(\varphi \right)x+I_{1}+I_{2}=0$, and the value of η is determined by the real root of the equation

(6) $$P\left(\eta \right)=\left(0.01k-1\right)\eta^{3}-2\eta^{2}+1+I_{1}+I_{2}=0$$

According to the Cardan discriminant, if there is an equation of $f\left(x\right)=ax^{3}+bx^{2}+cx+d=0$ and a≠0, then *p*, *q*, and Δ can be defined as

(7) $$\left\{\begin{array}{c} p=\frac{3ac-b^{2}}{3a^{2}}\\ q=\frac{27a^{2}d-9abc+2b^{3}}{27a^{3}}\\ \Delta =\frac{q^{2}}{4}+\frac{p^{3}}{27} \end{array}\right.$$

Then, for the AC-induced HR neuron model proposed in this paper,

(8) $$\left\{\begin{array}{c} p=\frac{-4}{3{\left(0.01k-1\right)}^{2}}\\ q=\frac{I_{1}+I_{2}+1}{0.01k-1}-\frac{16}{27{\left(0.01k-1\right)}^{3}} \end{array}\right.$$

Generally, there are three solutions for Equation (6), which can be given by

(9) $$\left\{\begin{array}{c} \eta_{1}=\sqrt[{3}]{-\frac{q}{2}+\sqrt{\Delta }}+\sqrt[{3}]{-\frac{q}{2}-\sqrt{\Delta }}+\frac{2}{3(0.01k-1)}\\ \eta_{2}=-\frac{1-\sqrt{3}i}{2}\sqrt[{3}]{-\frac{q}{2}+\sqrt{\Delta }}-\frac{1+\sqrt{3}i}{2}\sqrt[{3}]{-\frac{q}{2}-\sqrt{\Delta }}+\frac{2}{3(0.01k-1)}\\ \eta_{3}=-\frac{1+\sqrt{3}i}{2}\sqrt[{3}]{-\frac{q}{2}+\sqrt{\Delta }}-\frac{1-\sqrt{3}i}{2}\sqrt[{3}]{-\frac{q}{2}-\sqrt{\Delta }}+\frac{2}{3(0.01k-1)} \end{array}\right.$$

The discussion is divided into three situations. If Δ>0, there is only one real root foot for Equation (6). If Δ<0, there are three real root feet for Equation (6). If Δ=0, there are two real root feet for Equation (6) [31].

The Jacobian matrix at the equilibrium point *E* can be deduced as

(10) $$J_{E}=\left[\begin{array}{ccc} 6\eta -3\eta^{2}+0.01k\eta^{2} & 1 & 0.02k\eta^{2}\\ -10\eta & -1 & 0\\ 1 & 0 & -1 \end{array}\right]$$

The eigenvalues can be obtained by solving the characteristic polynomial

(11) $$P\left(\lambda \right)=\det\left(1\lambda -J_{E}\right)=\lambda^{3}+m_{1}\lambda^{2}+m_{2}\lambda +m_{3}=0$$

where

(12) $$\begin{array}{c} m_{1}=\frac{1}{100}\left(200-600\eta +300\eta^{2}-k\eta^{2}\right),\\ m_{2}=\frac{1}{100}\left(600\eta^{2}-4k\eta^{2}-200\eta +100\right),\\ m_{3}=\frac{1}{100}\left(400\eta +300\eta^{2}-3k\eta^{2}\right). \end{array}$$

**Figure 1:**
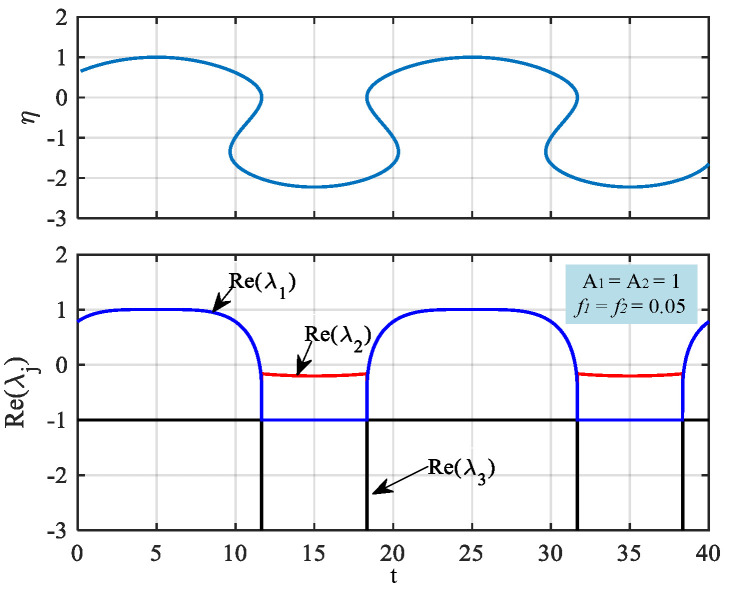
The η-values of the equilibrium point *E* and the corresponding eigenvalues $\lambda_{1},\lambda_{2},\lambda_{3}$, with the time evolutions.

The stability of the equilibrium point can be judged by the magnitude of λ. When the real parts of all eigenvalues λ are negative, the equilibrium point of the system is stable, and the solution of the system tends to the equilibrium point. When the real parts of all eigenvalues λ are positive, the equilibrium point is unstable and the solution of the system is far from the equilibrium point. When the real part of the eigenvalue λ is positive or negative, the equilibrium point is called the saddle point and is unstable. When *k* is set to 1, $A_{1}$ and $A_{2}$ are set to 1, and $f_{1}$ and $f_{2}$ are set to 0.05; Figure 1 shows the trajectory of the parameter η, the real part of the eigenvalues $\lambda_{1},\lambda_{2}$, and the partial real part of the eigenvalues $\lambda_{3}$ changing with time. It is obvious that, with the evolution of time, $\lambda_{1}$ jumps between positive and negative, resulting in the changing stability of the equilibrium. The stability of the equilibrium under these parameters during the whole time interval (5, 25) of a period of $I_{1}+I_{2}$ is discussed in Table 1 in detail. The type of equilibrium will change over time, which shows that the injected AC in the HR model can generate complex dynamic behaviors.

## 3. AC-Induced Complex Dynamical Behaviors under Single Excitation

In this section, when a single excitation $I_{1}$ is considered and $I_{2}$ is set to 0, the proposed model can exhibit complex dynamic behaviors which can be discussed in different aspects. It is worth mentioning that the MATLAB ODE45 algorithm is used to draw the phase diagrams, maximum Lyapunov exponent, bifurcation diagrams, and time evolutions.

Phase diagrams, bifurcation diagrams, and Lyapunov exponent diagrams are important tools in the study of chaos. The phase space structure of a chaotic system can be observed in the phase diagram, which can help acquire an in-depth understanding of the dynamic behavior of the system. In addition, the structure of the chaotic system is unstable, and any small appropriate disturbance will cause a sudden change in the topological structure of the system. This sudden change is called bifurcation, and we can observe the bifurcation situation of the system from the bifurcation diagram. The positive Lyapunov exponent is an important characteristic of a chaotic system. When the Lyapunov exponent is greater than 0, the system is chaotic.

### 3.1. Coexisting Asymmetric Firings When $A_{1}$ Changes

**Figure 2:**
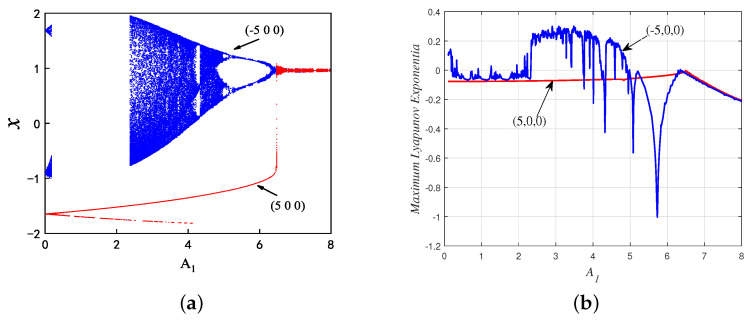
The improved memristor HR neuron model’s coexistence asymmetric attractor behavior for the initial values (−5,0,0) and (5,0,0) with respect to the following parameters $A_{1}$: (**a**) Bifurcation diagram; and (**b**) Maximum Lyapunov exponent.

Setting the parameter *k* as 1, and $f_{1}$ as 0.5, take $A_{1}$ as a bifurcation parameter within the range of (0, 8). The bifurcation diagram with respect to $A_{1}$ is numerically simulated by the MATLAB ODE45 algorithm in Figure 2a, in which (5, 0, 0) and (−5, 0, 0) are the initial values of the red and blue trajectories, respectively. And, the corresponding maximum Lyapunov exponent graph is shown in Figure 2b, which is consistent with the bifurcation diagram. As can be seen from Figure 2, the presented neuron model (3) shows a reverse period-doubling bifurcation under the initial condition of (−5, 0, 0), while the neuron model is always in a stable periodic state under the initial condition of (5, 0, 0).

**Figure 3:**
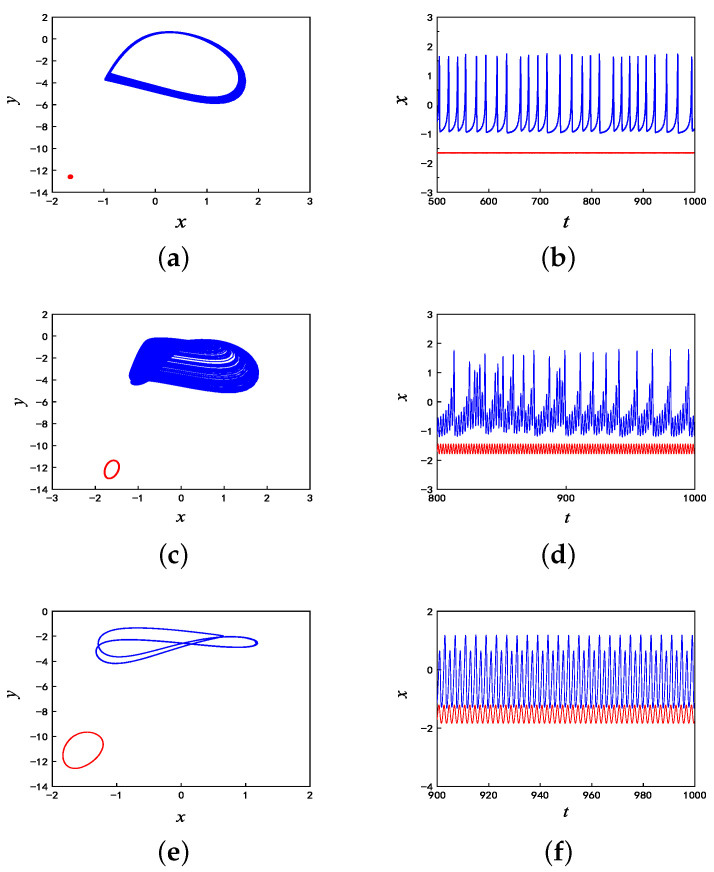
Coexisting firing patterns with different values of $A_{1}$: (**a**) Phase diagram when $A_{1}=0.1$; (**b**) Time sequences when $A_{1}=0.1$; (**c**) Phase diagram when $A_{1}=3$; (**d**) Time sequences when $A_{1}=3$; (**e**) Phase diagram when $A_{1}=5.5$; and (**f**) Time sequences when $A_{1}=5.5$.

Choose several representative values of $A_{1}$ to observe the coexisting behaviors of asymmetric firings. Then, the phase plane orbits corresponding to different values of $A_{1}$ are shown in Figure 3. When $A_{1}=0.1$, the time sequence exhibits a coexistence of fixed-point and chaotic spiking firing under the symmetric initial conditions of (−5, 0, 0) and (5, 0, 0). The corresponding phase diagram and time series are drawn in blue and red, which are shown in Figure 3a and Figure 3b, respectively. When $A_{1}=3$, as shown in Figure 3c,d, the coexistence of chaotic bursting firing with multiple spikes and periodic limit cycles is found in this improved HR model. It can be seen from Figure 3e,f that, when $A_{1}=5.5$, the coexistence of the periodic spiking firing and periodic limit cycles is found.

### 3.2. Coexisting Firing Patterns When $f_{1}$ Changes

**Figure 4:**
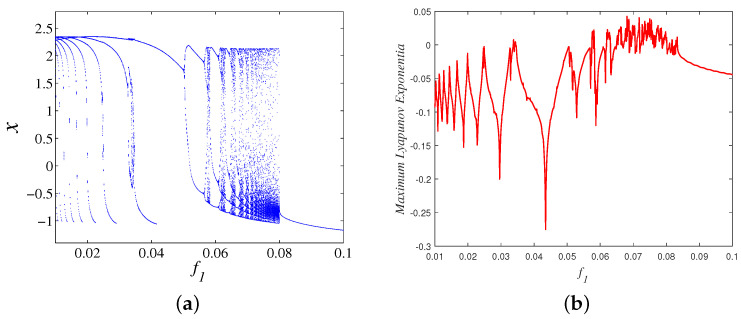
Bifurcation diagram and maximum Lyapunov exponent spectra with respect to $f_{1}$ in the range of (0.01, 0.1): (**a**) Bifurcation diagram; and (**b**) Maximum Lyapunov exponent.

**Figure 5:**
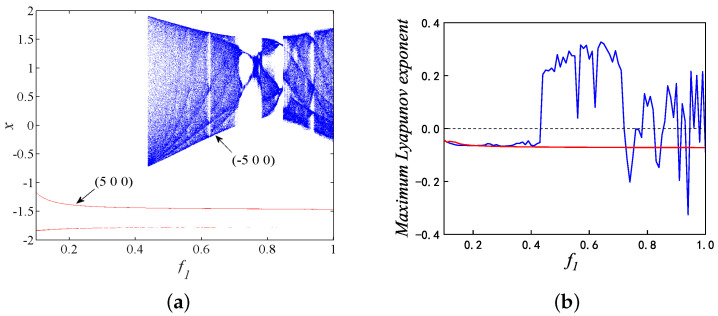
The improved memristor HR neuron model’s coexistence asymmetric attractor behavior for the initial values of (−5,0,0) and (5,0,0) with respect to the following parameters $f_{1}$: (**a**) Bifurcation diagram; and (**b**) Maximum Lyapunov exponent.

Set the parameter *k* as 1, $A_{1}$ as 3, take $f_{1}$ as a bifurcation parameter within the range of (0.01, 0.1), and the initial conditions are (−5, 0, 0). Then, the bifurcation diagram and maximum Lyapunov exponent are depicted in Figure 4a and Figure 4b, respectively. It can be seen from Figure 4 that the improved HR model has rich dynamic behaviors including chaos, period, periodic windows, crisis scenario, and so on. The maximum Lyapunov exponent is greater than zero. which means that the model is in a chaotic state. Obviously, the chaotic region is small in this range, and there is no coexistence phenomenon in this range. Then, a larger range is chosen, and when $f_{1}$ is in the range of (0.1, 1), the coexistence phenomenon appears in the partial region of (0.438, 1), and the bifurcation diagrams under different initial conditions are depicted in Figure 5a. In contrast to Figure 4, there is a larger chaotic range in Figure 5, and the maximum Lyapunov exponent spectra shown in Figure 5b is consistent with the bifurcation diagram.

### 3.3. Influence of Coupling Strength *k* on Dynamics

As we all know, multistability depends on the initial condition of the systems, and is a common phenomenon in dynamical systems. However, sometimes it is necessary to avoid multistability when designing commercial devices with certain special characteristics, which often brings an inconvenience to the design. In this study, through numerical simulation, it can be found that the coexistence phenomenon can be eliminated when *k* is less than −6.9. This is an interesting phenomenon which is rarely reported before. Details are discussed below.

**Figure 6:**
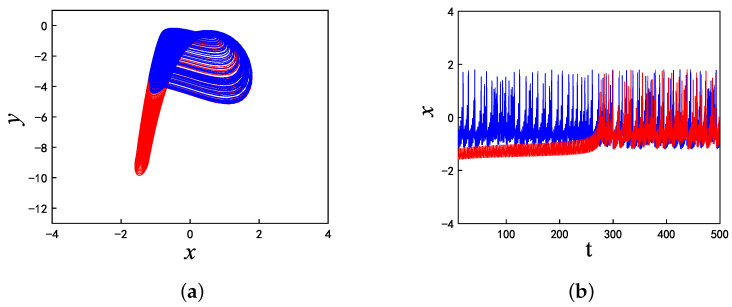
For the initial values (−5,0,0) (blue track) and (5,0,0) (red track), the coexisting firing patterns are eliminated at t=270 when k=−7: (**a**) phase diagram; and (**b**) time series.

**Figure 7:**
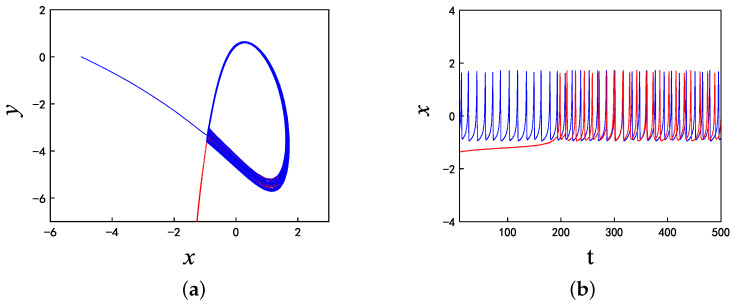
For initial values (−5,0,0) (blue track) and (5,0,0) (red track), the coexisting firing patterns are eliminated at t=200 when k=−10: (**a**) Phase diagram, (**b**) Time series.

When $A_{1}$ is equal to 3 and $f_{1}$ is equal to 0.5, set k=−7, for which the phase diagrams and corresponding time series are shown in Figure 6, where the initial condition of the blue track is (−5, 0, 0) and the initial condition of the red track is (5, 0, 0). It is evident that the blue trajectory is in a state of chaotic bursting fire during the whole time range, while the red trajectory enters into chaotic bursting fire from the periodic orbit when *t* is approximately equal to 270. Similarly, when k=−10, and $f_{1}=0.5$, $A_{1}=0.1$, as shown in Figure 7, the blue trajectory is always in a state of spiking while the red trajectory enters into the spiking state after *t* = 200. It can be seen from these two sets of parameters that the memristor coupling strength *k* can eliminate the coexistence phenomenon with the time evolution when *k* is less than −6.9 and the proposed neuron model will eventually enter into a stable state under any initial conditions. This phenomenon makes a great contribution to multistability control.

## 4. Different Firing Patterns Are Driven by High–Low-Frequency Current

The application of two frequency signals is rife in neural systems, and it plays a significant role in the biological field, such as that the neural cell behaviors under high–low-frequency ultrasonic irradiation can be examined. Then, the dynamical behaviors under high–low-frequency current are worth discussing. In this section, $f_{1}$ is regarded as a high-frequency electromagnetic radiation, and $f_{2}$ is regarded as a low-frequency electromagnetic radiation. When k=1, $A_{1}=A_{2}=3$, and $f_{1}=0.5$, the initial condition is (−5, 0, 0), with the increase in low frequency $f_{2}$, the electrical activities of the HR neuron are investigated in Table 2. When $f_{2}=0.002$, the model presents a periodic bursting firing pattern with chaotic spiking per bursting, and when $f_{2}=0.02$, there is a periodic bursting firing pattern with three spikes per bursting. When $f_{2}=0.04$, a period-2 spiking pattern is found, and the model presents a chaotic spiking pattern when $f_{2}=0.07$. The three corresponding three Lyapunov exponents are also calculated in Table 2. Obviously, the quiescent state between the two bursts becomes shorter as the frequency increases. These different mode transitions can be achieved by adjusting the value of $f_{2}$.

## 5. Circuit Implementation

To prove that multiple firing patterns of the HR neuron model under the multi-frequency AC injection can be realized in hardware circuits, we carry out circuit implementation. In this section, the circuit schematic will be analyzed and designed. The amplifiers and the multipliers are selected as UA741 and AD633JN. They are both under the supply voltage ±15 V. And, the gain of the multiplier is 0.1. In order to complete the circuit implementation, the following transformation is taken out. Let $\tau =\tau_{0}t$ where the integral constant $\tau_{0}$ is the time-scaling factor and $\tau_{0}=\frac{1}{RC}=$ 10,000. Set C=50 nF, then *R* can be solved as 2 kΩ.

**Figure 8:**
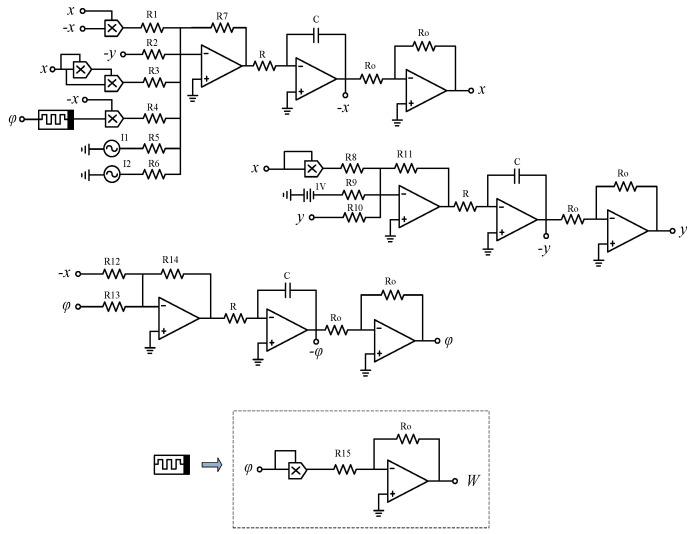
Circuit implementation of the improved memristive HR neuron model.

To verify the MATLAB simulation results, the circuit schematic was designed in Figure 8. When *k* = 1, the circuit equivalent equation derivation process is:

1. Variable-scale reduction transformation. Since the range of the attractor does not exceed the dynamic range of ±13 V, variable-scale reduction transformation of variety is not required.
2. Time-scale transformation:
  (13) $$\left\{\begin{array}{c} \frac{dx}{d(\tau_{0}t)}=\frac{R_{7}}{10R_{1}}x^{2}+\frac{R_{7}}{R_{2}}y-\frac{R_{7}}{100R_{3}}x^{3}+\frac{R_{7}}{10R_{4}}W\left(\varphi \right)x+\frac{R_{7}}{R_{5}}I_{1}+\frac{R_{7}}{R_{6}}I_{2}\\ \frac{dy}{d(\tau_{0}t)}=\frac{R_{11}}{R_{9}}-\frac{R_{11}}{10R_{8}}x^{2}-\frac{R_{11}}{R_{10}}y\\ \frac{d\varphi }{d(\tau_{0}t)}=\frac{R_{14}}{R_{12}}x-\frac{R_{14}}{R_{13}}\varphi \end{array}\right.$$
3. Differential–integral conversion:
  (14) $$\left\{\begin{array}{c} x=\frac{1}{RC}\int [\frac{R_{7}}{10R_{1}}x^{2}+\frac{R_{7}}{R_{2}}y-\frac{R_{7}}{100R_{3}}x^{3}+\frac{R_{7}}{10R_{4}}W\left(\varphi \right)x+\frac{R_{7}}{R_{5}}I_{1}+\frac{R_{7}}{R_{6}}I_{2}]dt\\ y=\frac{1}{RC}\int [\frac{R_{11}}{R_{9}}-\frac{R_{11}}{10R_{8}}x^{2}-\frac{R_{11}}{R_{10}}y]dt\\ \varphi =\frac{1}{RC}\int [\frac{R_{14}}{R_{12}}x-\frac{R_{14}}{R_{13}}\varphi ]dt \end{array}\right.$$
4. Because the inverse addition proportional arithmetic unit is used in the circuit, the Equation (14) is normalized to:
  (15) $$\left\{\begin{array}{c} x=\frac{1}{RC}\int [\frac{R_{7}}{10R_{1}}(-x)x+\frac{R_{7}}{R_{2}}\left(-y\right)-\frac{R_{7}}{100R_{3}}x^{3}+\frac{R_{7}}{10R_{4}}W\left(\varphi \right)\left(-x\right)+\frac{R_{7}}{R_{5}}I_{1}+\frac{R_{7}}{R_{6}}I_{2}]dt\\ y=\frac{1}{RC}\int [\frac{R_{11}}{R_{9}}-\frac{R_{11}}{10R_{8}}x^{2}-\frac{R_{11}}{R_{10}}y]dt\\ \varphi =\frac{1}{RC}\int [\frac{R_{14}}{R_{12}}\left(-x\right)-\frac{R_{14}}{R_{13}}\varphi ]dt \end{array}\right.$$
  where the memductance function equivalent circuit is
  (16) $$W\left(\varphi \right)=\frac{R_{o}}{10R_{15}}\varphi^{2}$$

In order to make the values of resistances as integral as possible, set $R_{7}$ = 300 kΩ and $R_{11}=R_{14}$ = 100 kΩ. Then, by comparing the corresponding coefficients in Equation (15) with Equation (3), all the values of the circuit parameters can be obtained in Table 3 in detail. Finally, the multisim simulation is constructed based on the component parameters above.

**Figure 9:**
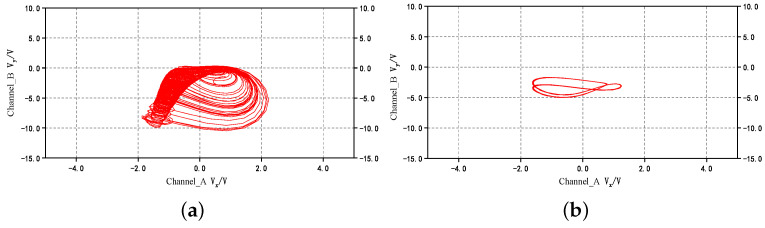
Phase diagram of circuit simulation: (**a**) Chaotic bursting; and (**b**) Periodic spiking.

**Figure 10:**
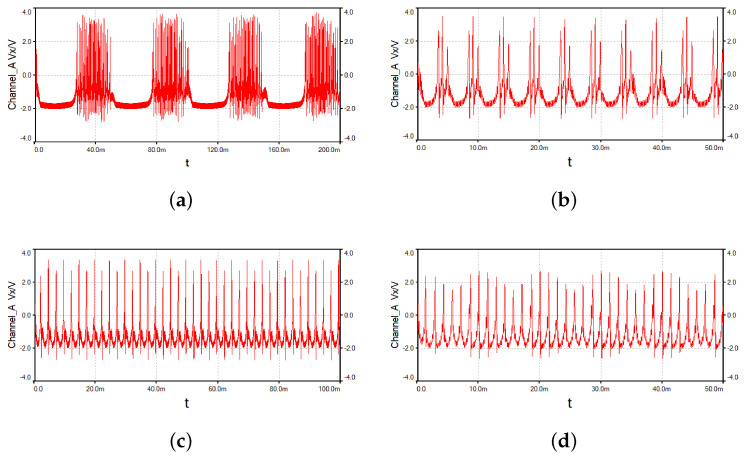
Time series under different $f_{2}$: (**a**) $f_{2}$ = 20 Hz; (**b**) $f_{2}$ = 200 Hz; (**c**) $f_{2}$ = 400 Hz; and (**d**) $f_{2}$ = 700 Hz.

When only the external current $I_{1}$ is connected to the circuit, and the amplitude $A_{1}$ is 3 and 5.5, respectively, the frequency $f_{1}$ is equal to 5 kHz. Then the corresponding phase diagrams in the oscilloscope are shown in Figure 9. When $A_{1}$ is equal to 3, a chaotic bursting firing pattern is obtained, and when $A_{1}$ is equal to 5.5, a periodic spiking firing pattern is obtained which is completely consistent with the numerical simulation in Figure 3. The circuit of two external currents is also constructed. Set the amplitudes $A_{1}=A_{2}=3$ and $f_{1}$ as 5 kHz, then adjust the value of $f_{2}$ to 20 Hz, 200 Hz, 400 Hz, and 700 Hz. The time series in the oscilloscope are shown in Figure 10. These are a periodic bursting fire pattern with chaotic spiking per bursting; a periodic bursting fire pattern with periodic-3 spiking per bursting; a periodic-2 spiking firing pattern; and a chaotic spiking firing pattern. The numerical simulation results are verified accurately through analog circuit simulation.

## 6. Conclusions

In this paper, we constructed an improved memristive HR neuron model with multi-frequency AC injection. Then, the equilibrium of the model is analyzed. When the model is under single excitation, it can produce the coexistence of different firing patterns. With the introduction of the memristor, it can be found that the multistability of the model is eliminated when the memristor coupling strength *k* is less than −6.9. Additionally, when the external current is a high–low frequency excitation, the system can exhibit different kinds of firing patterns under the changing low frequency. Finally, the circuit experiment is carried out to verify the validity of the numerical simulation results.

## Figures and Tables

**Table 1:** The equilibrium point *E*, three eigenvalues, and its stability for $A_{1}=A_{2}=1$, $f_{1}=f_{2}=0.05$.

t	Equilibrium	$\lambda_{1},\lambda_{2}$ and $\lambda_{3}$	Stability
5,25	(1, −4, 1)	$\lambda_{1,2}=1.005\pm 2.4413i$, $\lambda_{3}=-1$	Unstable
11.64,18.34	(−2.02, −19.402, −2.02)	$\lambda_{1}=-0.1505$, $\lambda_{2}=-25.1599$, $\lambda_{3}=-1$	Stable
15	(−2.22, −23.642, −2.22)	$\lambda_{1}=-0.1995$, $\lambda_{2}=-28.8564$, $\lambda_{3}=-1$	Stable

**Table 2:** Different firing patterns under low frequency $f_{2}$.

LEs	$f_{2}$	Firing Patterns
LE1 = −0.0536 LE2 = −1.0056 LE3 = −16.7675	$f_{2}$ = 0.002	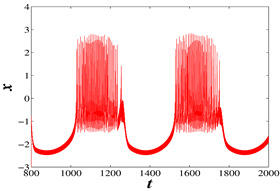
LE1 = −0.1018 LE2 = −1.0062 LE3 = −17.9204	$f_{2}$ = 0.02	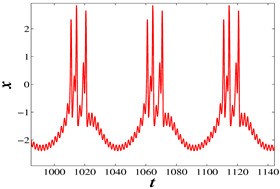
LE1 = −0.0129 LE2 = −1.0062 LE3 = −16.8427	$f_{2}$ = 0.04	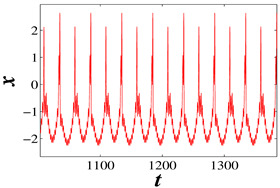
LE1 = 0.0276 LE2 = −1.0065 LE3 = −14.0263	$f_{2}$ = 0.07	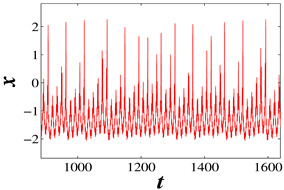

**Table 3:** Circuit parameters of the improved HR neuron model.

Parameters	Significations	Value
*R*, $R_{8}$	Resistance	2 kΩ
$R_{3}$	Resistance	3 kΩ
Ro, $R_{1}$	Resistance	10 kΩ
$R_{4}$	Resistance	30 kΩ
$R_{9}$, $R_{10}$, $R_{11}$, $R_{12}$, $R_{13}$, $R_{14}$, $R_{15}$	Resistance	100 kΩ
$R_{2}$, $R_{5}$, $R_{6}$, $R_{7}$	Resistance	300 kΩ
*C*	Capacitor	50 nF

## Data Availability

Data are contained within the article.
